# Use of a Novel Resistive Strain Sensor Approach in an Experimental and Theoretical Study Concerning Large Spherical Storage Tank Structure Behavior During Its Operational Life and Pressure Tests

**DOI:** 10.3390/s20020525

**Published:** 2020-01-17

**Authors:** Virgil Florescu, Stefan Mocanu, Laurentiu Rece, Robert Ursache, Nicolae Goga, Constantin Viorel Marian

**Affiliations:** 1 Technological Equipment Faculty, Mechanical Technology Department, Technical University of Civil Engineering of Bucharest, RO-020396 Bucharest, Romania; mocanustef@gmail.com (S.M.); rece@utcb.ro (L.R.); robionut@gmail.com (R.U.); 2 Foreign Languages Engineering Department, University Politehnica Bucharest, RO-060042 Bucharest, Romania; n.goga@rug.nl (N.G.); constantinvmarian@gmail.com (C.V.M.)

**Keywords:** resistive strain sensor, large pressurized spherical tank, FEM, structure optimization

## Abstract

This paper introduces a new method for the use of tensor-resistive sensors in large spherical storage tank equipment (over 12,000-mm diameters). We did an experiment with 19 petroleum or ammonia product sphere-shaped storage tanks with volumes of 1000 and 1800 cubic meters, respectively. The existing literature only contains experiments based on sensors for tanks with diameters no larger than 600 mm. Based on a number of resistive strain sensor measurements on large spherical pressurized vessels regarding structural integrity assessment, the present paper is focused on the comparison between "real-life" obtained sensor data versus finite element method (FEM) simulation results. The present paper is structured in three parts and examines innovative directions: the use of the classic tensor-resistive sensors in a new approach concerning large structural equipment; an original 3D modeling method with the help of the FEM; and conclusions with possible implications on the regulations, design, or maintenance as a result of the attempt of mutual validation of the new methods previously mentioned.

## 1. Introduction

Experimental structural strain-field assessment by means of a resistive strain sensor approach and finite element method (FEM)-based optimization concerning the geometrical positioning of resistive strain sensors are the subject of many research studies [1,2,3,4,5,6,7,8,9,10]. Moreover, the study of large, spherical, storage-specific tank structures becomes a real challenge due to geometric particularities. To the best of our knowledge, the existing literature contains only experiments based on sensors for tank equipment with diameters no larger than 600 mm. Liquid petroleum gas (LPG)- and ammonia-filled spherical tanks (used for our sensors measurements) are critical equipment. Their failure (cracks, explosions, etc.) can lead to massive losses in terms of human life and goods.

In practice, such tanks are periodically tested while functioning to check their operating condition. The investigation and assessment of current equipment are performed for safety purposes. In accordance with the law, the pressure vessels are subjected for periodically inspection. A test consists of checking whether the thin wall of the vessel is being subjected to an overpressure (about 1.25 times the maximum authorized operating pressure) and remains in the elastic domain [11,12,13,14]. Because the test procedure requires the measurement of the internal pressure at the top of the sphere, due to the large diameter of the structure, the water column additionally strains the mantle in the bottom area with an extra 0.12 to 0.16 MPa; for some cases, this represents an overstrain of about 15–20%. The problem becomes even more complex when one needs to estimate the remaining service life of the installation according to existing regulations. As noted above, such tanks are critical equipment and it is important to test them for proper functioning, especially in the light of newer findings. For example, as reported in the paper, the stairs (which are not nationally or internationally regulated as constructive parts of large pressure vessels) may interfere with the proper functioning of the tanks.

Most of the studies carried out in this field have focused on the pipe-type structures used with measurements performed by means of bidirectional tensor-resistive sensors [2,3], or studies carried out under laboratory conditions [4,5,6,7]. For example, Agbo et al. presented a study [1] that describes investigations performed using tensor-resistive sensors regarding the behavior of some thin-walled metal structures with an operating usage history and subjected to an internal pressure. In comparison with these laboratory studies, and from the point of view of both FEM simulations and the experimental approach, this paper addresses large spherical structures that require in situ treatment and the use of three-direction sensors.

Regarding the way in which the thickness of the wall was treated in the FEM from the meshing point of view, Zhu et al. [5] approached the problem of resistance and stability by applying FEM on some spherical structures; however, they focused only on the manhole area, while the experiment was performed on some laboratory models (experimental verification using acrylonitrile butadiene styrene (ABS) scale models). In the case of the FEM application for small structures (several millimeters in size), the meshing operation does not raise particular problems in terms of the size or shape of the meshing element, alleviating the need for heavy computing resources. For a structure with dimensions of approximately 1.5 mm^2^, Messina et al. [7] used a meshing network with 250,000 elements. As compared with his work (and other similar ones), in our case, considering the physical size of the structure discussed in this paper (diameter between 12,000–16,000 mm with wall thicknesses between 20–45 mm), applying the FEM and obtaining useful and verifiable results proved to be a real challenge. As a starting point, we used the optimization method for single- and multi-axis load cell structures developed by Takezawa et al. [6], which was adapted to the complexity of the structures that were the subject of this study. This also led to the establishment of a new original problem-solving approach that implied the customization of the 3D modeling algorithm consisting of ten slice modules for large structures.

The present study proposed a comparative study of the results obtained by applying the FEM and the results obtained following resistive strain sensor measurements during in situ overpressure tests. The results obtained by applying the FEM according to the original problem-solving approach were useful both for finding the position of the resistive strain sensors for the experimental study, as it has been done in a similar way in References [8,9], and for the theoretical/analytical determinations of the specific values for the state of the tension and deformation (stress/strain field values). What makes our research original is the use of SolidWorks algorithmic symmetry for the FEM 3D modeling and simulations for large structures. To the best of our knowledge, this slicing modeling technique is unheard of in the scientific literature for complex and large structures.

The paper is structured as follows: Section 2 describes the material and methods used, Section 3 provides the results and discussions, and Section 4 shows the conclusions.

## 2. Methods, Materials, and Means of Investigation

The geometrical parameters, the working fluid, and the test pressure value for all 19 sphere tanks that were investigated and analyzed are given in Table 1.

This article presents the results obtained (and their comparison) by using two methods: in situ determination, namely stress assessment by applying resistive strain sensor measurements during overpressure tests under actual loads, and a theoretical one, by means of FEM.

The determination of the tension and deformation state of the strained bodies can be done analytically (by means of analytical calculation and/or numerical methods) or experimentally [15,16,17].

For the theoretical determination of tension and deformation state, the acceptance of simplifying hypotheses is required regarding the shape and structure of the element, the mechanical features of the material the element is made of, and/or its loading and support scheme. Moreover, in the above-mentioned papers, the material of the element on which the calculations are performed is considered ideal: continuous, homogeneous, isotropic, and perfectly elastic. In reality, these conditions are not fully met because of real conditions concerning technological and engineering processes [18,19]. In the case of bodies or elements with a more complicated geometrical shape and loading scheme, analytical calculation with numerical methods is quite difficult and requires the input of a trained operator for this purpose, as well as a prudent use of simplifying hypotheses.

For the FEM, results that are shown below were measured for sphere no. 8 (position 8 in Table 1), which was filled with LPG and had a volume of 1000 m^3^, a working pressure of 1 MPa, and a test pressure of 1.3 MPa. For all other spheres, results are similar.

According to the manufacturing documentation, the spherical tank was made of structural steel with the minimum mechanical features, which were as follows (Table 2), as recorded for one of the ferrules that make up the mantle:

The design is in accordance with EN 13445 [11,12]. Consequently, the determination of the admissible stress (noted f) is defined as the minimum value between the ratios Rc20/1.5 and Rm/2.4, where Rc20 is the tensile strength at 20 °C and Rm is the yield strength in MPa.

As a result of the EN 13445-3 calculus specification, the minimum required thickness for spherical shells depends on nominal design stress (f).

The normal design stress (f) is defined as:

f = min (Rp/1.5; Rm/2.4) where Rp is the yield strength and Rm is the tensile strength.

f = min (380/1.5; 560/2.4) = 233.33 MPa.

This value was also used as an input parameter for the FEM approach. For further reading regarding the FEM mathematical apparatus, we refer the reader to References [1,5,20]. General information regarding the FEM computational approach is given in the Supplementary Materials.

For experimental evaluation, the method involved finding the right resistive strain sensors and the identification of the most appropriate areas to make the measurements from, along with documentation and validation of the most probable perilous areas. To find the right areas, one method was to compute the maximum stressed areas of a structure (in our case a tank) through FEM 3D modeling and simulation.

The authors’ initial purpose was to compare the experimental results measured with the simulated values using the FEM. However, unexpected results were obtained. Anticipating further discussions, our paper reveals the fact that the access stairs do influence the mechanical behavior of the tanks. No regulations at the national or international level consider the effect of the stairs. To the best of our knowledge, this result is not reported in the literature. According to the results, our recommendation is that access stairs should be considered by existing regulations related to the mechanical behavior of the tanks. This is further detailed in the next sections.

## 3. Results and Discussion

### 3.1. FEM Simulations

FEM simulations have both qualitative and quantitative components. The qualitative component consists in the identification of the most stressed areas, which determines the scheme of the sensors’ arrangement. The quantitative component consists in estimating the value field concerning the primary strain analytical calculations.

In the FEM analysis, the following simplifying hypotheses were considered: the thickness of the material was uniform for the entire thin wall of the sphere and the thickness of the wall was the smallest value measured using an ultrasound technique.

The software used for modeling and simulation was Dassault Systèmes SolidWorks 2014 (Watham, MA, USA) [21].

It is a well-known fact that SolidWorks represents a complex software tool for design and simulation purposes; to emphasize this aspect, one can quote J. Ed Akin’s work, *Finite Element Analysis Concepts via SolidWorks, 2009*: SolidWorks “studies calculate displacements, reaction forces, strains, stresses, failure criterion, factor of safety and error estimates. Available loading conditions include point, line, surface and thermal loads, elastic orthotropic materials are also available. The SW Simulation software also offers several types of nonlinear studies” [20].

**Figure 1:**
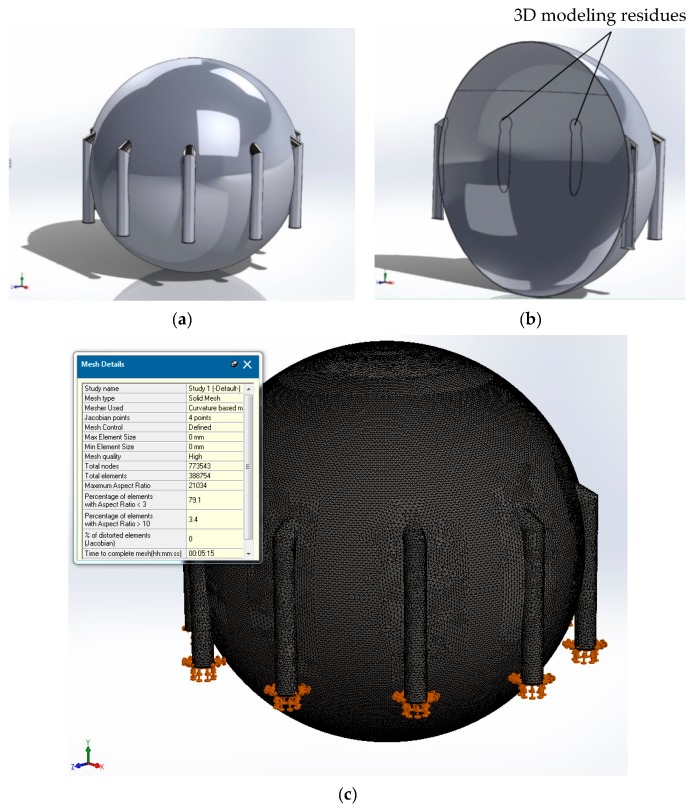
3D model of full “part” option simulation parameters: (**a**) tank simulation model using the “part” option, (**b**) tank simulation modeling issues using the “part” option, and (**c**) 3D modeling using the unique study element finite element method (FEM) meshing with 388,754 elements for a single full part case.

**Figure 2:**
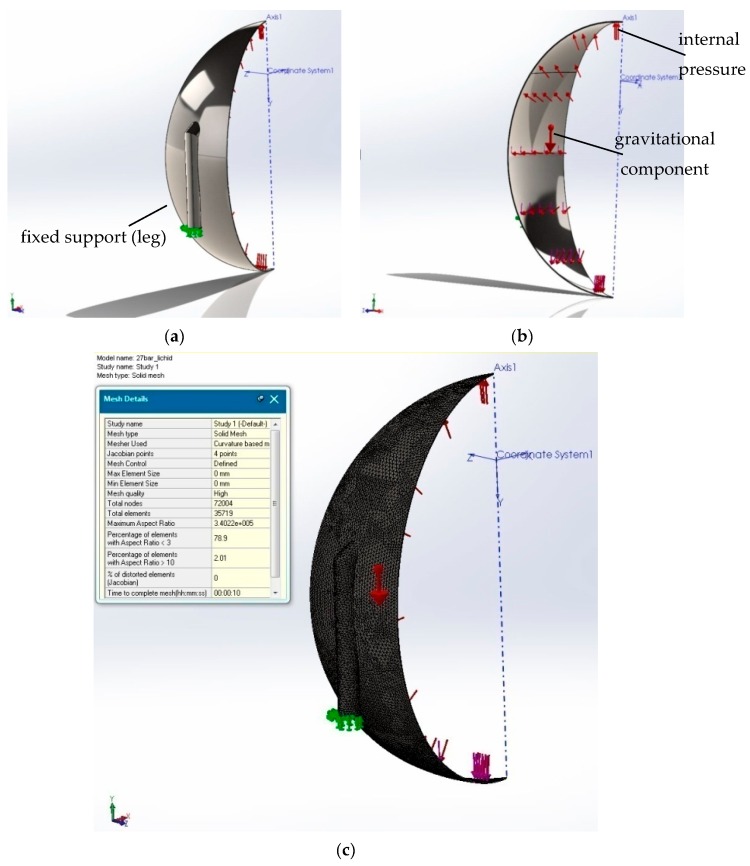
3D model of the “slice part” option simulation parameters: (**a**) SolidWorks representation rules concerning the boundary conditions in terms of the degrees of freedom, (**b**) SolidWorks representation rules concerning the boundary conditions in terms of the loading parameters, and (**c**) 3D modeling using symmetry FEM meshing with 35,719 elements for each slice part.

Application of SolidWorks Simulation 2014 allows for several approaches for 3D modeling and simulation:

(1) A first criterion for the selection of meshing is the use of the “part” type elements (individual parts) or “assembly” (entity made of several individual parts) (see Figure 1 and Figure 2).

(2) Second, a simplification can be made such that the simulation time is shortened and less computational resources are used by using the symmetry option (circular, in this case) when 3D modeling and meshing the element that is going to be studied.

For our mesh tank simulations, by using a single element (using option “part” without the use of symmetry), one can obtain the model in Figure 1a. Some of the existing disadvantages are the impossibility of choosing different materials for the elements, namely the spherical tank and its support legs, respectively. Another disadvantage is the existence of some “residues” (see Figure 1b), as seen on the transversal section of the element from Figure 1a; one can observe the traces of the intersection of the legs of the spherical tank with the tank itself (in the center). We call these traces 3D modeling residues; these residues will cause problems related to the application of the FEM method (with implications for the difficulty of meshing and, implicitly, for the degree of accuracy).

As a first measure for raising the accuracy of the treatment, one can employ 3D modeling with the use of SolidWorks symmetry. This method allows for the choice of several variants, among which is the algorithmic treatment with the help of circular symmetry, which we consider to be of maximum efficiency in our case in terms of the number of meshing elements used and the computational simulation time. The circular symmetry allowed us to use just a slice (Figure 2a) of the tank element for meshing and simulation (as represented in Figure 1c) from a total number of ten slices in which the tank was divided. The input data of the simulation are presented in Figure 2b,c. The results obtained in this case were expected to be closer to the real values because this method allowed fora larger number of finite elements because the modeled slice portion was smaller compared to the one for the whole tank from Figure 1a.

According to SolidWorks representation rules (Figure 2a,b), different red-sized arrows stand for forces or pressure fields and green custom arrows and indicators stand for boundary conditions in terms of degrees of freedom (displacements) as supporting scheme.

In Figure 2b, the loads coming from internal pressure, weight of the contained LPG, and weight of the tank itself are represented. In Figure 2c, one can notice the local coordinate system, denoted Coordinate System1 (Figure 2c), in relation to which the law of linear variation of the load could be modeled according to the actual weight of the LPG stored in the liquid phase by considering an 80% full container (according to the working manual instructions, a tank will be filled only 80% from the total volume, where the rest should remain empty for vaporization phenomena). The FEM parameters are represented in Figure 2c.

**Figure 3:**
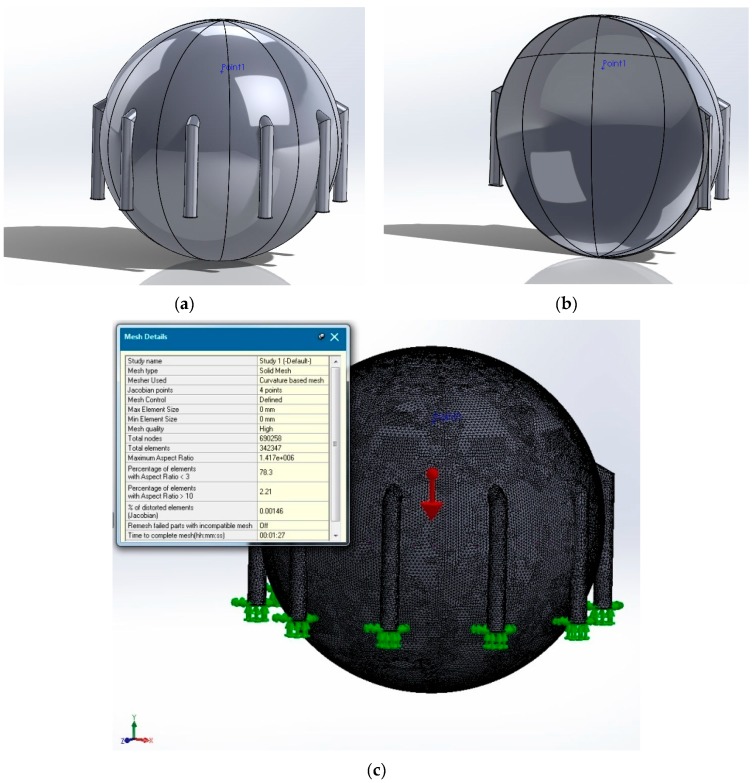
3D modeling through an “assembly” set consisting of ten slice modules: (**a**) tank simulation model using the “assembly” option, (**b**) better 3D modeling quality with the absence of issues using the “assembly” option, and (**c**) mesh elements using a ten-slice modular assembly.

We performed simulations with one slice as represented in Figure 2a–c. In order to validate and confirm the results obtained with one slice, we used also the option of “assembly” for meshing and simulation. In this case, we used the whole set of ten slices without using the method of simplifying the calculation scheme by using symmetry (see Figure 2). As noted in Figure 2c and Figure 3c, the mesh grid execution time for one slice with options “part” and “symmetry” was approximately 10 s compared to 1.5 min for an assembly composed often slice modules. Moreover, one can see (Figure 3b) a better 3D modeling quality (as compared to Figure 1b) due to the total absence of residual artifacts.

The use of the SolidWorks symmetry algorithm for the FEM 3D modeling and simulations of the large structures considered makes this work original; to the best of our knowledge, for complex and large structures, this slicing modeling technique is unheard of in the scientific literature.

**Figure 4:**
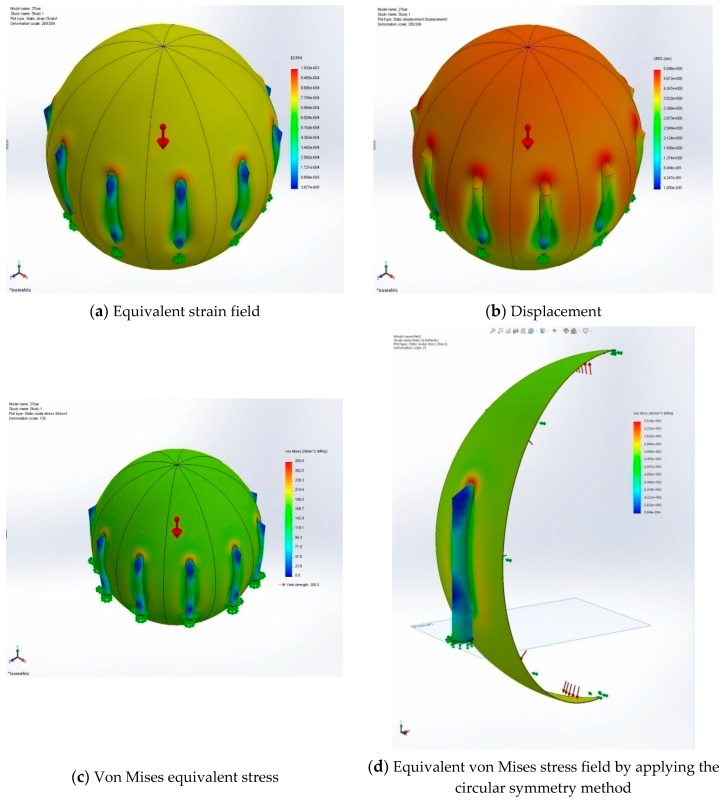
(**a**) The equivalent strain field distribution. It can be seen that the most problematic stress area of the tank (zone with color red in the figure) was the place where the supporting legs were linked to the main tank. This gives the points where the resistive sensors were placed for the experimental data acquisition. (**b**) The actual displacements of the tank (in mm) are represented. As expected, the zones of largest displacements (color red in the figure) are also (as in (**a**)) the places where the supporting legs were linked to the main tank. (**c**) The von Mises equivalent stress is given. These values were compared to the theoretical ones. In what follows we will describe the results of FEM simulations for the option “part’ with the application of the circular symmetry method. From the whole set of generated data, we present only the equivalent von Mises stress field ones (**d**), where similar conclusions can be drawn for the rest of the results. When compared with (**c**), it can be seen that the von Mises distributions were similar and the range values were equivalent. This validated the fact that using just one slice with this circular symmetry option was a sufficient replacement for the whole FEM computations.

The results of the FEM simulations (option “assembly”) are: the values of the equivalent strain field (Figure 4a), the displacements components field (Figure 4b), the von Mises equivalent stresses (Figure 4c), and the maximum von Mises equivalent stresses field (Figure 4d). In the mechanics of materials, the von Mises yield criterion (also known as the maximum distortion energy criterion) can be formulated in terms of the von Mises stress or equivalent (von Mises) tensile stress, which represents a computable scalar value of stress; a material is said to start yielding when the von Mises stress reaches a value known as the yield strength. The von Mises stress is used to predict the yielding of materials under complex loading from the results of uniaxial tensile stress. Based on a known plane stress state defined by principal normal stresses $\sigma_{1},\sigma_{2},$ the equivalent von Mises stress is given as Equation (1):

(1) $$\sigma_{von\;Mises}=\sqrt{\sigma_{1}^{2}+\sigma_{2}^{2}-\sigma_{1}\sigma_{2}}.$$

### 3.2. Experimental Method

In what follows, we present the results for the experimental method.

Using the FEM simulations, we determined the zones (the area of intersection of tank with supporting legs) where the resistive strain sensors were to be placed. Using the sensors mounted in the determined places, we obtained the experimental data. In this section, we compare the theoretical von Mises equivalent stress obtained using the FEM method with the experimental ones.

The resistive strain sensor electro-resistive measurements method (measuring electrical resistance variations undergone in a strain gauge sensor grid linked to specific resistive strain sensor bridge equipment) represents one of the most frequently used experimental techniques and targets the real behavior of the material of the mechanical structure that is being investigated. The option to apply this method is based on the fact that research can be carried out on the structure under actual operating conditions.

It is well known that for materials situated in the elastic behavior limit (Hooke’s theory), there is a linear relationship between specific deformations (strains) and stresses. Above this limit, plastic deformation occurs and the relation between the specific deformations and stresses is no longer linear; moreover, the equations that express the stress/strain connection become very complex.

Any specific structural element deforms under a load, i.e., the existence of a normal/tangential field stress (σ, τ). Experimental direct calculation of such tensile parameters is impossible; therefore, in order to address the issue, one can experimentally determine the corresponding strain field and then, based on theoretical relationships (Hooke’s theory) between specific deformations and stresses, establish the stress field values.

The main disadvantage of the experimental method resides in the fact that it does not directly identify the most strained areas of the equipment, a situation that may be overcome with the help of the previous FEM study.

**Figure 5:**
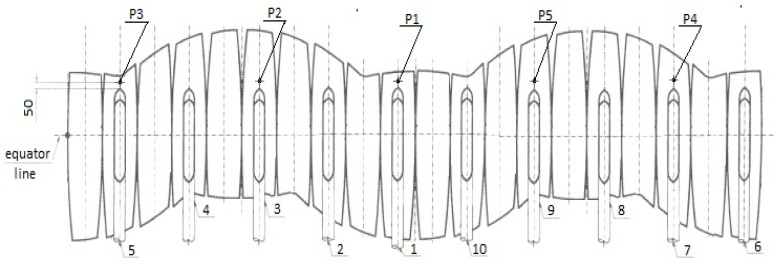
The plan for the resistive strain sensor positions.

Starting from the geometrical and constructive particularities of the tank, and in order to be able to determine the von Mises stress field state, it has been decided that for each measuring point, a three-directional strain sensor (rosette type) should be used (three-directional strain sensors give the strains according to three directions and this can be used to characterize the principal stresses and their directions; other types of sensors are not adequate for this). The location plan of the resistive strain sensor is shown in Figure 5.

In Figure 5, the original constructive design of the tank is presented together with the sensor placements. As observed in the figure, the constructive design is also formed by slices that are not correlated with the ones from the theoretical FEM simulation (the only common part is that both slices include the area of intersection between the sustaining legs and the tank). In Figure 5, it can be observed that only five sensors were used. This was due to regulatory measurements rules for such tanks and due to the consideration of costs. The policy was to first place a smaller number of sensors, and only if abnormal irregularities were observed, more sensors would be deployed on the tank and the measurements would be repeated. In our case, five sensors were considered enough to be used for the first series of measurements. As determined from the FEM simulations sensors were placed in the area of the intersection between the legs and the tank (as seen in Figure 5).

**Figure 6:**
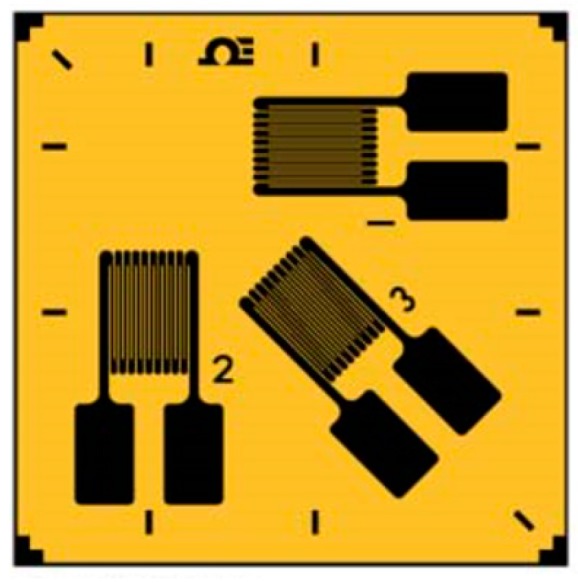
Three-directional strain sensor.

**Figure 7:**
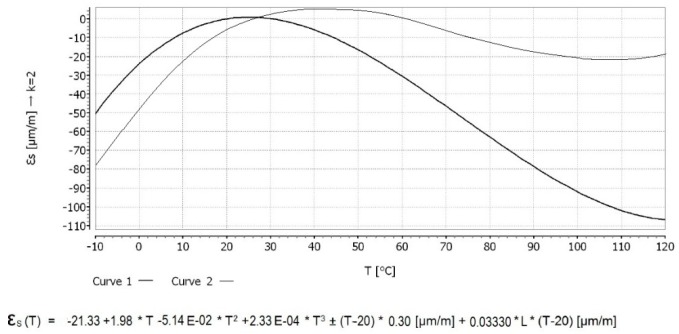
Three-directional strain sensor characteristics.

In order to perform load-related strain field assessment, a rosette-type sensor with three measurement directions (Figure 6) was used, with the corresponding temperature characteristics shown in Figure 7. The sensor manufacturer recommends the use of temperature correction curve 2 from Figure 7 for a HBM 6/350 CRY81-3L-3M-type sensor (Hottinger Baldwin Messtechnik Gmbh, Darmstadt, Germany).

### 3.3. Comparison Between the FEM Simulations and Experimental Results

Table 3 indicates the sensor-recorded values (equivalent von Mises stress) with respect to the most strained/stressed point of the supporting legs. The first five columns correspond to the five points from Figure 5, while the sixth column corresponds to the FEM-calculated values. Characteristics of the nineteen tanks are given in Table 1. The calculated FEM stress from column 5 was the one obtained with the “assembly” option. Given the symmetry, the stress was the same for all ten FEM slices; therefore, we put just one value in Table 3. Because we did both with the “assembly” and “part” options, we took the highest stress value from the two FEM simulations to emulate a worst-case scenario; for all cases, although the values were similar, the highest values were given by FEM simulations with the “assembly” option.

From Table 3, it can be observed that FEM values and experimental values for points P2, P4, and P5 were similar, while P1 and P3 were not. For validating this statistically, we used Tukey’s test. Through this test, which verifies the equality of the means for “*k*” selections of possibly different volumes, we aimed to identify the similarities and differences between the values measured experimentally for each supporting leg and the value calculated using the FEM.

The means ${\bar{X}}_{j}$ were calculated using the Equation (2), which determines ${\bar{X}}_{\min}$ and ${\bar{X}}_{\max}$:

(2) $${\bar{X}}_{j},j=\bar{1},\bar{k},$$

where “*k*” is the number of different volumes involved and *j* the number of selected measurement points. 

The formula for Tukey’s test is:

(3) $$q=\frac{{\bar{X}}_{\max}-{\bar{X}}_{\min}}{SE},$$

where ${\bar{X}}_{\max}$ is the larger of the two means being compared, ${\bar{X}}_{\min}$ is the smaller of the two means being compared, and *SE* is the standard error of the sum of the means, which is calculated using Equation (4):

(4) $$SE=\sqrt{\frac{S_{1}^{2}+S_{2}^{2}}{n}},$$

where *S*_1_^2^ and *S*_2_^2^ are the dispersions for the two corresponding selections ${\bar{X}}_{\min}$ and ${\bar{X}}_{\max}$.

Appendix A contains the raw data and auxiliary information.

The computed “*q*” value was compared to a certain value obtained from the standardized range distribution “*q_a_*” for n–k degrees of freedom, where n = 19, the total number of investigated equipment, and k = 2. One can consider *q_a_* to be the critical value. It was considered that the values were different if the computed *q* value was greater than the critical value *q_a_*.

The results are presented in the Table 4.

The values obtained for *q_a_* with 17 degrees of freedom are presented in Table 5.

Using Tukey’s test, it was concluded that P2, P4, and P5 value were consistent with the FEM analysis. A significant difference was observed for the values corresponding to points P1 and P3 in relation to the FEM-calculated value. In all cases, the most stressed point was P1, which corresponded to the position of the leg attached to the tank access stairs. Accordingly, the less stressed point was the diametrically opposed one, which was P3 in all cases. Excluding the most stressed point (P1) and the least stressed point (P3), it can be seen that the values for P3, P4, and P5 measured using the tensor-resistive sensors in three directions were within the margin of ±3% compared to the values calculated using the FEM method. This was quite remarkable and doubly validates both the FEM simulations and the experimental method (one validates another).

The difference between the values recorded by sensors at P1 (adjacent to the tank stair access structure) and the opposite one (P3) was quite significant, with this phenomenon being systematically reported in all cases of the investigated spheres. As noted, P1 was the most stressed point while P3 was the least stressed. The access tank stairs was adjacent to P1, placed between P5 and P1. In this context, P3 can be considered the opposite point to P1 (and not also P4), where the position of the access stairs explains why P3 was the less stressed (and not also P4). One result of the study is the fact that the presence of stairs causes a peak stress value point at P1. Such an unconventional structural behavior was caused by specific interface areas (between the stairs and tank sphere) that are prone to stress concentration due to a sudden local increase in terms of general mechanical stiffness caused by the stair’s presence, with a corresponding undesired influence from the point of view of the mechanics of materials.

Hence, from the point of view of the general stress state field, the sensor data in Table 2 indicate an unacceptable trend concerning the structural fatigue strength (in point P1) with poor life expectancy characteristics.

One can ask why we did not model the stair structures using the FEM. Our starting point for FEM modeling was the European regulatory norms, as defined in EN 13445 (similar in USA and worldwide), which is particular to our tank spheres. This regulation does not include the stairs in the mechanical design of the sphere; therefore, we did not include the stairs in the FEM modeling. However, the experimental results show clearly that the stairs had an influence on the stress behavior of the tank; however this, as noted, is not regulated. We will enlarge this discussion in the next section.

## 4. Conclusions

Based on the observations from the experimental data and the FEM, we argue that there is a need for a new algorithmic convergence from the design, manufacture, and maintenance perspectives. The study revealed areas that, during periodic tests, could reach the flow limit as a result of initial constructive solutions.

Based on the experimental results recorded for the 19 spheres subjected to resistive strain sensor measurements (electrical resistance variations undergone in a rosette-type three-directional sensor grid linked to a specific resistive strain sensor bridge equipment) obtained using periodic technical state evaluation of spherical thin-walled pressure vessels, a notable difference between the values obtained using 3D modeling via finite element computing (Figure 4c,d) and in situ resistive strain sensor readings were highlighted at point P1 next to the tank access stairs. An approximately 30% higher strain/stress was observed due to the leg supporting the stairs. The tank access stairs are not regulated internationally (for example in EU regulatory document EN 13445), but according to the sensor measurements, they heavily influence the mechanical stress behaviors with important consequences regarding potential failures (cracks, explosions, etc.). Our research leads to the following recommendations:

(1) Regarding attached linked stairs, these must be considered in the regulatory documents for the structural behavior (such as EU regulation EN 13445).

(2) Possible solutions can be: changing the position (relocation) of the supporting structure of the access stairs in the case of the classical projects with a large number of operating hours (for example to periodically change the stairs from one position to another around the sphere tank), which is a measure that can be combined with the modification of an access stair support scheme, especially for newly designed tanks, by using technical solutions that would result in an increase of the number of degrees of freedom of the support stair scheme (for example, “joint”-type support point network).

Another conclusion is that, excluding the least stressed points (P3) and the most stressed ones (P1), the theoretical FEM results and the experimental ones obtained through the measurements usingthe rosette-type three-direction sensors were very similar. This shows once more that such sensor measurements are very reliable and useful for the mechanical behavior assessment concerning large structural tanks.

## Figures and Tables

**Table 1:** Main features of the spheres that were investigated in this study. LPG: liquid petroleum gas.

No.	Volume (m^3^)	Diameter (mm)	Working Fluid	Working Pressure (MPa)	Test Pressure (MPa)	Manufacture Year
1	1800	15,100	LPG	1	1.3	1984
2	1000	12,400	LPG	1	1.3	1982
3	1000	12,400	LPG	0.5	0.6	1981
4	1000	12,400	LPG	1	1.3	1980
5	1000	12,400	LPG	0.6	0.6	1981
6	1800	15,100	LPG	1	1.3	1983
7	1000	12,400	LPG	0.6	0.6	1981
8	1000	12,400	LPG	1	1.3	1983
9	1800	15,100	LPG	1	1.3	1981
10	1800	15,100	LPG	1	1.3	1980
11	1800	15,100	LPG	2.65	2.65	1983
12	1000	12,400	LPG	1	1.3	1980
13	1000	12,400	LPG	0.8	0.8	1979
14	1000	12,400	LPG	0.8	0.8	1979
15	1000	12,400	LPG	0.8	0.8	1979
16	1000	12,400	Gasoline	2.4	3	1978
17	1000	12,400	Gasoline	2.4	3	1979
18	1000	12,400	NH_3_	2.1	2.7	1964
19	1000	12,400	NH_3_	2.1	2.7	1964

**Table 2:** Mantle material properties.

Material Properties	Value
Tensile strength at 20 °C temperature	560 MPa
Yield threshold at specified temperature	380 MPa

**Table 3:** Determined values for the most strained/stressed points.

Sphere	Maximum Stress Per Supporting Leg (MPa)					Calculated Stress	Overload Per Leg Relative to the Most Stressed Leg (in All Cases P1) %			
	P1	P2	P3	P4	P5	FEM	P2	P3	P4	P5
1	169	124	110	125	128	136	36.29	53.63	35.2	32.03
2	181	144	109	138	140	145	25.69	66.05	31.16	29.29
3	185	121	107	127	132	125	52.89	72.89	45.67	40.15
4	208	162	113	156	159	170	28.40	84.07	33.33	30.82
5	104	86	65	82	85	90	20.93	60.00	26.83	22.35
6	221	152	124	142	149	154	45.39	78.22	55.63	48.32
7	296	229	171	226	233	239	29.25	77.17	46.84	27.03
8	331	255	168	244	251	253	29.80	42.59	32.76	31.87
9	202	151	130	144	149	155	33.77	55.38	40.28	35.57
10	213	166	133	152	163	165	28.31	60.15	40.13	30.67
11	322	261	193	252	258	165	23.37	66.83	27.78	24.81
12	284	208	151	181	216	210	36.54	88.07	56.91	31.48
13	199	143	104	128	136	145	39.16	91.34	55.47	46.32
14	208	155	109	141	149	155	34.19	90.82	47.52	39.60
15	164	128	94	116	121	130	28.13	74.46	41.38	35.54
16	323	253	206	236	249	255	27.27	56.31	36.44	29.32
17	299	244	192	219	231	255	22.54	55.73	36.53	29.44
18	169	126	102	118	123	128	34.13	65.69	43.22	37.40
19	159	116	99	109	120	128	37.07	60.60	45.87	32.50

**Table 4:** The values calculated using Tukey’s test.

	P1	P2	P3	P4	P5	FEM
**Means** ${\bar{X}}_{j}$	223	169.6842	130.5263	159.7895	168	168.5789
***SE***	4368.889	2965.45	1513.152	2600.064	2853.778	2465.368
***q***	2.869446	0.065375	2.629667	0.538309	0.034601	

**Table 5:** Calculated *q_a_* values.

Threshold	10%	5%	1%
***q_a_***	1.333	1.74	2.11

**Figure A1:**
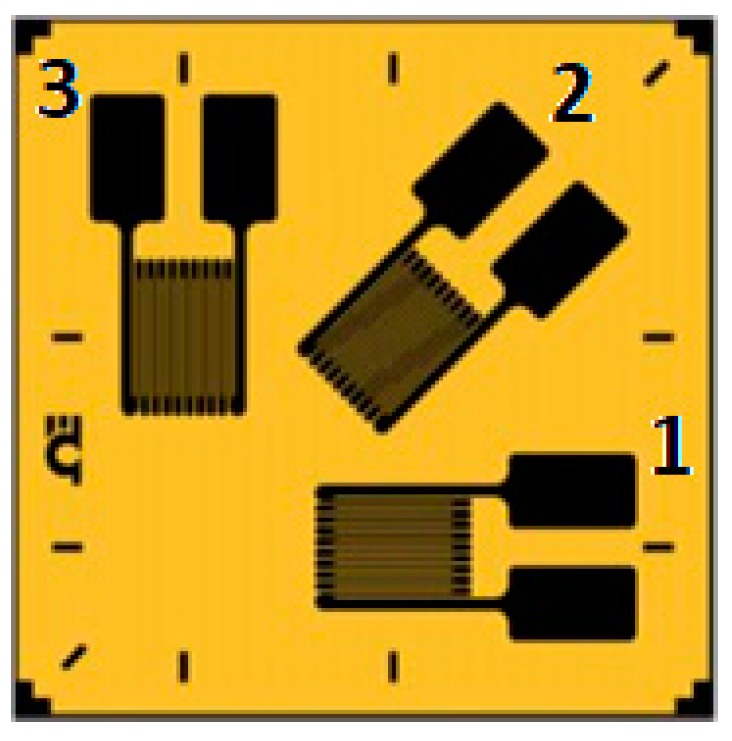
Three directional grid sensors.

## Supplementary Materials

The following are available online at https://www.mdpi.com/1424-8220/20/2/525/s1.

## Appendix A

In order to perform load related strain field assessment, a rosette type sensor with three measurement directions (Figure A1) has been used.

The sensor type is HBM 6/350 CRY81-3L-3M. The grid counting was done according to Figure A1, grid 1 being horizontal.

Raw data aquistion was made by means of QuantumX MX1615B strain gauge bridge. One bridge amplifier channel was allocated for each grid. There for supporting leg one (P1) the channels 1,2,3 were allocated, for the second one (P2) 4,5,6, and so on until (P5) 13,14,15.

Table A1 contains the maximum test pressure raw data recorded for storage tank no.8, Table 1 from the main paper. The increasing and decreasing ramp values are not included.

**Table A1:** Raw data.

P1 Channel (µm/m)			P2 Channel (µm/m)			P3 Channel (µm/m)			P4 Channel (µm/m)			P5 Channel (µm/m)		
1	2	3	4	5	6	7	8	9	10	11	12	13	14	15
989.5276	935.9271	941.2488	778.9627	636.6916	707.6031	535.6578	498.2269	448.672	753.86	645.8469	671.5721	768.527	731.2713	711.8824
998.8672	955.2855	955.0379	781.2703	645.6916	717.9694	537.2447	505.2696	455.2449	756.0932	654.9763	681.4104	770.8037	741.6082	722.3113
1014.147	951.0137	968.9021	791.3178	648.2627	728.3921	544.1539	507.2816	461.8537	765.8169	657.5844	691.3024	780.7165	744.5613	732.797
1024.458	982.3405	975.6859	799.3632	654.6601	733.4919	549.6863	512.2877	465.0874	773.603	664.0738	696.1426	788.6541	751.909	737.9277
1041.292	989.0687	983.5378	812.4985	666.8393	739.3947	558.7189	521.8182	468.8302	786.3151	679.4281	701.7448	801.6135	765.8974	743.8662
1043.855	993.0047	982.4747	814.4984	660.5843	738.5955	560.0941	523.9235	468.3234	788.2505	680.0832	700.9863	803.5866	768.7132	743.0622
1048.754	998.2721	983.7043	818.321	673.1274	732.0022	562.7228	526.7799	466.1428	791.95	682.8067	694.7287	807.358	773.1196	736.429
1046.138	997.0032	981.0702	816.2797	672.7951	730.022	561.3191	526.4788	462.8872	789.9744	682.4696	698.8493	808.344	772.7379	741.4368
1041.663	992.5443	989.2973	812.7879	669.231	736.2068	564.9179	523.6898	466.8088	786.5951	680.8542	698.7193	807.899	772.6443	740.6591
1042.334	996.769	984.5232	809.3232	664.4755	732.6178	562.5354	525.9685	464.5331	793.2421	680.0303	695.313	807.4807	771.1824	737.0483
1044.056	999.3265	992.6892	814.6552	662.539	738.7568	560.2019	524.4532	468.4257	788.4022	678.066	701.1394	809.7412	769.9583	743.2244
1043.207	992.9122	990.2144	813.9928	669.4841	736.8963	559.7464	523.8879	467.246	787.7612	679.111	699.3736	809.0877	770.9351	741.3527
1041.181	993.2855	992.641	808.4235	669.765	738.7206	561.9167	524.1077	468.4027	792.3713	679.3959	701.105	809.593	769.2577	743.188
1050.887	996.0382	984.0992	819.9855	665.5899	732.2991	563.8674	525.8406	470.331	793.5608	680.1608	695.0105	809.0002	771.4624	742.7276
1049.21	995.8983	981.5763	818.6767	663.8832	730.4025	562.9674	524.505	469.1284	792.2941	678.4295	693.2105	807.7089	771.5021	740.8196
1049.578	993.2925	982.3521	818.9637	663.4492	730.9857	563.1647	524.1654	469.4982	792.5719	677.9892	693.7639	807.9921	771.0037	741.4063
1042.688	990.3609	991.2728	813.5881	660.316	737.692	559.4682	521.7136	467.7505	787.3696	674.8111	700.1288	807.6885	770.4051	742.1532
1045.626	983.9285	995.0075	815.8804	663.3022	740.4996	561.0444	524.0504	469.5308	789.5879	677.8402	702.7935	809.95	770.8349	744.9778
1034.126	990.5992	993.5343	806.9067	662.4839	739.3921	559.8736	523.41	468.8285	790.9034	677.0101	701.7423	808.0966	769.895	743.8636
1042.875	994.3786	989.5178	813.7335	669.6522	736.3726	564.5682	524.0194	466.9139	787.5103	679.2814	698.8766	809.8319	770.1281	743.8258
1038.208	999.8489	987.8519	810.0918	668.1827	735.1202	562.0639	522.8695	466.1198	792.9859	677.7909	697.688	808.239	772.4404	742.5659
1039.989	995.3378	987.5572	811.4821	658.0268	734.8987	563.0199	521.9222	465.9794	785.3314	677.4889	697.4777	809.6106	773.7758	742.343
1034.565	991.0989	994.8091	816.3935	662.2217	740.3505	561.3973	522.2048	469.4362	790.0845	678.7441	702.6519	809.4563	770.5938	744.8277
1036.471	996.4632	996.3457	815.9165	664.2675	741.5056	561.0693	523.8057	470.1686	789.6229	673.8193	703.7482	808.9857	770.9435	745.9899
1037.549	992.5354	994.3338	818.7475	663.2012	739.9932	563.016	522.9714	469.2096	792.3627	677.7377	702.3128	807.7787	770.7189	744.4683
1048.032	997.9596	990.4449	812.17	666.6305	737.0696	558.493	525.6548	467.3559	791.9971	679.2163	699.5381	809.2894	770.6576	744.527
1043.034	996.5329	997.0272	821.0833	671.1177	742.0179	564.62	525.1662	470.4935	793.6232	680.768	704.2345	809.3832	770.8113	746.5053
1047.805	994.5519	1000.385	814.7395	659.1209	744.0419	563.2599	523.7784	470.7768	788.4838	678.5987	705.7554	807.8244	769.0325	748.5415
1047.183	996.8727	991.2118	813.9197	659.0731	737.6461	563.6962	523.741	467.7214	794.6232	678.5502	700.0853	807.0157	768.9775	742.107
1052.295	996.2634	989.8161	820.4882	665.0301	744.1146	564.2131	520.4025	471.8229	794.0473	677.5929	704.9243	809.4961	772.8194	744.8345
1044.687	993.5956	985.5638	814.5828	659.1148	740.9178	562.1522	523.7736	469.7959	788.3322	682.864	703.1904	807.6699	771.0254	745.3985
1045.557	993.6773	983.4446	816.2789	662.1862	739.3246	561.3185	526.1771	468.7857	789.9736	677.7081	701.6783	809.3433	769.5531	743.7957
1042.983	993.1266	985.1959	814.9686	670.4237	740.6412	560.4174	524.6231	469.6206	788.7055	680.0641	702.9279	808.0504	770.0143	745.1203
1047.098	996.5665	987.2407	816.2344	673.1813	742.1785	561.2879	526.781	470.5953	789.9306	682.3613	704.3868	810.0822	772.6215	746.6668
1044.447	998.35	986.2634	815.7936	671.9631	741.4438	560.9848	525.8278	470.1294	789.504	681.6256	703.6895	809.8645	771.7824	745.9277
1044.373	988.0768	982.75	814.9113	666.2529	731.2848	560.3781	521.3593	463.6879	788.6501	675.8333	706.2191	808.994	772.2239	748.612
1039.496	994.1367	988.2458	811.4526	663.5961	735.4164	561.9997	524.2803	466.3076	792.3029	673.1383	702.9691	805.5816	769.1724	746.8638
1048.225	994.4183	987.1122	818.7739	663.7859	734.5641	563.0342	524.4289	465.7672	792.3882	673.3309	702.1602	809.8048	769.3905	746.0064
1037.519	993.2181	986.3153	808.3941	662.9767	733.9651	562.8965	523.7957	465.3874	792.3429	672.51	701.5917	808.5641	768.461	745.4037
1042.927	991.4882	986.3183	820.9991	661.8102	733.9673	564.5644	522.8829	465.3888	794.5417	671.3268	701.5938	810.0002	773.1817	745.406
1040.771	993.0337	986.4957	817.6685	662.8524	734.1007	562.274	523.6984	465.4734	791.3184	672.3838	701.7204	806.7142	771.3182	745.5402
1038.557	991.1228	986.1499	819.66	661.5638	733.8408	563.6436	522.6901	465.3086	793.2458	671.0768	701.4737	808.6791	769.8383	745.2787
1046.116	990.5983	983.8195	820.3218	660.1397	732.0888	564.0986	521.5757	464.1977	793.8862	669.6322	699.8109	809.3319	770.2026	743.5161
1048.156	993.7983	983.4135	819.8569	660.6528	731.7836	563.779	521.9772	464.0042	793.4363	670.1527	699.5213	808.8733	770.7919	743.2091
1051.958	998.0074	987.668	815.9821	660.1375	734.982	561.1144	521.5739	466.0322	789.6863	669.6299	702.5568	808.0504	770.2	746.4268
1047.041	997.8248	983.994	816.6648	661.5254	732.22	561.5839	522.66	464.2809	790.3471	671.0378	699.9354	808.7239	771.7941	743.6481
1047.051	994.3475	984.447	818.9857	661.0273	732.5605	563.1798	522.2702	464.4968	792.5931	670.5326	700.2586	808.0137	771.2221	743.9907
1036.55	991.3914	988.4474	818.0624	664.997	737.8232	562.5449	525.3766	467.8337	791.6996	674.5593	705.2534	807.1028	771.7814	742.2852
1048.2	993.8306	987.0224	818.1546	669.4819	742.0143	562.6083	523.8862	470.4912	791.7889	679.1088	702.2311	807.1938	768.9326	746.5017
1043.373	989.6468	986.1201	814.1066	666.4332	738.8163	563.8247	526.5004	468.4634	787.8714	676.0162	703.1959	808.2001	772.4309	743.2843
1036.684	981.899	985.7533	810.8047	662.7673	734.2485	561.5541	523.6318	465.5671	790.6758	672.2975	696.8606	808.9423	768.2205	738.6888
1041.513	997.3636	980.8068	813.873	665.666	737.3417	563.6641	525.9001	467.5284	793.6452	675.238	699.7963	807.9695	771.5498	741.8007
1042.057	995.6747	983.2304	813.3169	666.6539	739.1636	563.2817	526.6732	468.6836	793.1071	676.2401	701.5255	807.4209	772.6845	743.6337
1049.5	997.0154	980.0701	811.7232	665.5372	736.7878	562.1858	525.7993	467.1772	791.5647	675.1073	699.2707	805.8486	771.4018	741.2436
1046.301	992.3239	988.9767	817.456	672.3121	743.4836	562.1279	526.1008	471.8228	791.1128	681.9796	705.6255	809.5045	772.1831	747.9798
1038.44	992.7368	983.8779	804.1224	670.1579	739.6504	560.959	524.4151	468.9923	792.2088	679.7944	701.9875	808.3496	769.709	744.1234
1038.954	997.6335	988.5264	810.6282	671.8664	735.6273	562.4328	525.7521	466.4414	794.5051	681.5275	698.1693	807.7683	771.6713	746.076
1037.058	984.3716	986.3013	809.3804	663.0452	733.9545	561.5747	525.8493	465.3807	793.2975	672.5795	696.5816	808.5372	770.5397	744.3931
1035.02	991.5296	989.7854	802.5648	664.1273	736.5738	560.8879	526.6961	467.0415	791.7015	673.6771	699.0675	809.8129	771.7826	747.0282
1040.078	983.6	980.2905	811.9161	663.0762	736.9536	563.3184	525.8735	467.2823	791.7514	672.6109	699.428	808.0388	770.5753	747.4103
1040.694	994.7924	988.0233	812.2441	663.3316	735.2491	563.5439	526.0734	466.2015	792.0688	672.87	697.8103	808.3625	770.8686	745.6955
1038.065	988.5565	986.9288	816.0203	663.8447	734.4263	561.1406	526.4749	465.6798	795.7233	673.3905	697.0294	809.088	771.458	744.8677
1040.596	997.6662	989.1539	811.9206	665.0858	736.0991	562.3215	527.4461	466.7405	791.7558	674.6494	698.617	808.0433	772.8834	746.5506
1045.505	990.9	987.6176	817.7657	662.0861	742.4619	562.3409	525.0987	470.775	791.4125	671.6065	704.6558	809.8101	769.4381	746.9519
1044.893	988.1161	985.3714	815.3084	658.7885	740.7732	560.6511	522.5183	469.7042	792.0344	678.2615	703.0531	807.3857	771.6506	745.253
1041.539	987.1783	986.751	812.6914	659.2814	741.8103	558.8515	522.904	470.3618	789.5017	672.7615	704.0374	807.8038	772.2167	746.2964
1042.614	995.8895	982.9047	813.5301	659.1458	738.9188	559.4283	522.7979	468.5284	790.3134	673.6239	701.2932	808.6312	772.061	743.3874
1039.277	993.4449	984.4047	810.9259	665.0338	740.0464	557.6375	525.4054	469.2434	787.7931	674.5967	702.3634	808.0619	772.8237	744.5219
1046.13	993.0779	987.0914	816.2737	663.5712	742.0662	561.3149	524.2609	470.5241	792.9686	673.1131	704.2803	808.3381	771.1439	746.5539
1036.646	999.0153	984.2942	808.8734	666.0036	738.9484	556.226	526.1643	468.5472	790.8067	675.5804	701.3213	807.0369	772.9376	743.4172
1040.864	993.8702	986.8982	805.7831	664.1115	742.7218	554.101	522.6837	470.9398	791.8161	673.6611	704.9025	805.9881	770.7644	747.2134
1036.112	987.9839	982.9955	808.4567	666.6471	739.4555	553.9395	521.6678	468.8687	782.4035	674.2332	701.8025	797.6258	765.6766	743.9274
1005.809	957.2608	936.0034	784.812	644.4017	702.3225	539.6801	504.2602	445.3237	759.5207	653.6679	666.5603	774.2978	740.1267	706.5698
990.4138	913.5164	909.4469	772.7993	621.7312	690.0476	531.4195	486.52	437.5405	747.8952	630.6714	654.9105	762.4461	714.0886	694.2207
989.5276	935.9271	941.2488	778.9627	636.6916	707.6031	535.6578	498.2269	448.672	753.86	645.8469	671.5721	768.527	731.2713	711.8824
998.8672	955.2855	955.0379	781.2703	645.6916	717.9694	537.2447	505.2696	455.2449	756.0932	654.9763	681.4104	770.8037	741.6082	722.3113
1014.147	951.0137	968.9021	791.3178	648.2627	728.3921	544.1539	507.2816	461.8537	765.8169	657.5844	691.3024	780.7165	744.5613	732.797
1024.458	982.3405	975.6859	799.3632	654.6601	733.4919	549.6863	512.2877	465.0874	773.603	664.0738	696.1426	788.6541	751.909	737.9277
1041.292	989.0687	983.5378	812.4985	666.8393	739.3947	558.7189	521.8182	468.8302	786.3151	679.4281	701.7448	801.6135	765.8974	743.8662
1043.855	993.0047	982.4747	814.4984	660.5843	738.5955	560.0941	523.9235	468.3234	788.2505	680.0832	700.9863	803.5866	768.7132	743.0622
1048.754	998.2721	983.7043	818.321	673.1274	732.0022	562.7228	526.7799	466.1428	791.95	682.8067	694.7287	807.358	773.1196	736.429
1046.138	997.0032	981.0702	816.2797	672.7951	730.022	561.3191	526.4788	462.8872	789.9744	682.4696	698.8493	808.344	772.7379	741.4368
1041.663	992.5443	989.2973	812.7879	669.231	736.2068	564.9179	523.6898	466.8088	786.5951	680.8542	698.7193	807.899	772.6443	740.6591
1042.334	996.769	984.5232	809.3232	664.4755	732.6178	562.5354	525.9685	464.5331	793.2421	680.0303	695.313	807.4807	771.1824	737.0483
1044.056	999.3265	992.6892	814.6552	662.539	738.7568	560.2019	524.4532	468.4257	788.4022	678.066	701.1394	809.7412	769.9583	743.2244
1043.207	992.9122	990.2144	813.9928	669.4841	736.8963	559.7464	523.8879	467.246	787.7612	679.111	699.3736	809.0877	770.9351	741.3527
1041.181	993.2855	992.641	808.4235	669.765	738.7206	561.9167	524.1077	468.4027	792.3713	679.3959	701.105	809.593	769.2577	743.188
1050.887	996.0382	984.0992	819.9855	665.5899	732.2991	563.8674	525.8406	470.331	793.5608	680.1608	695.0105	809.0002	771.4624	742.7276
1049.21	995.8983	981.5763	818.6767	663.8832	730.4025	562.9674	524.505	469.1284	792.2941	678.4295	693.2105	807.7089	771.5021	740.8196
1049.578	993.2925	982.3521	818.9637	663.4492	730.9857	563.1647	524.1654	469.4982	792.5719	677.9892	693.7639	807.9921	771.0037	741.4063
1042.688	990.3609	991.2728	813.5881	660.316	737.692	559.4682	521.7136	467.7505	787.3696	674.8111	700.1288	807.6885	770.4051	742.1532
1045.626	983.9285	995.0075	815.8804	663.3022	740.4996	561.0444	524.0504	469.5308	789.5879	677.8402	702.7935	809.95	770.8349	744.9778
1034.126	990.5992	993.5343	806.9067	662.4839	739.3921	559.8736	523.41	468.8285	790.9034	677.0101	701.7423	808.0966	769.895	743.8636
1042.875	994.3786	989.5178	813.7335	669.6522	736.3726	564.5682	524.0194	466.9139	787.5103	679.2814	698.8766	809.8319	770.1281	743.8258
1038.208	999.8489	987.8519	810.0918	668.1827	735.1202	562.0639	522.8695	466.1198	792.9859	677.7909	697.688	808.239	772.4404	742.5659
1039.989	995.3378	987.5572	811.4821	658.0268	734.8987	563.0199	521.9222	465.9794	785.3314	677.4889	697.4777	809.6106	773.7758	742.343
1034.565	991.0989	994.8091	816.3935	662.2217	740.3505	561.3973	522.2048	469.4362	790.0845	678.7441	702.6519	809.4563	770.5938	744.8277
1036.471	996.4632	996.3457	815.9165	664.2675	741.5056	561.0693	523.8057	470.1686	789.6229	673.8193	703.7482	808.9857	770.9435	745.9899
1037.549	992.5354	994.3338	818.7475	663.2012	739.9932	563.016	522.9714	469.2096	792.3627	677.7377	702.3128	807.7787	770.7189	744.4683
1048.032	997.9596	990.4449	812.17	666.6305	737.0696	558.493	525.6548	467.3559	791.9971	679.2163	699.5381	809.2894	770.6576	744.527
1043.034	996.5329	997.0272	821.0833	671.1177	742.0179	564.62	525.1662	470.4935	793.6232	680.768	704.2345	809.3832	770.8113	746.5053
1047.805	994.5519	1000.385	814.7395	659.1209	744.0419	563.2599	523.7784	470.7768	788.4838	678.5987	705.7554	807.8244	769.0325	748.5415
1047.183	996.8727	991.2118	813.9197	659.0731	737.6461	563.6962	523.741	467.7214	794.6232	678.5502	700.0853	807.0157	768.9775	742.107
1052.295	996.2634	989.8161	820.4882	665.0301	744.1146	564.2131	520.4025	471.8229	794.0473	677.5929	704.9243	809.4961	772.8194	744.8345
1044.687	993.5956	985.5638	814.5828	659.1148	740.9178	562.1522	523.7736	469.7959	788.3322	682.864	703.1904	807.6699	771.0254	745.3985
1045.557	993.6773	983.4446	816.2789	662.1862	739.3246	561.3185	526.1771	468.7857	789.9736	677.7081	701.6783	809.3433	769.5531	743.7957
1042.983	993.1266	985.1959	814.9686	670.4237	740.6412	560.4174	524.6231	469.6206	788.7055	680.0641	702.9279	808.0504	770.0143	745.1203
1047.098	996.5665	987.2407	816.2344	673.1813	742.1785	561.2879	526.781	470.5953	789.9306	682.3613	704.3868	810.0822	772.6215	746.6668
1044.447	998.35	986.2634	815.7936	671.9631	741.4438	560.9848	525.8278	470.1294	789.504	681.6256	703.6895	809.8645	771.7824	745.9277
1044.373	988.0768	982.75	814.9113	666.2529	731.2848	560.3781	521.3593	463.6879	788.6501	675.8333	706.2191	808.994	772.2239	748.612
1039.496	994.1367	988.2458	811.4526	663.5961	735.4164	561.9997	524.2803	466.3076	792.3029	673.1383	702.9691	805.5816	769.1724	746.8638
1048.225	994.4183	987.1122	818.7739	663.7859	734.5641	563.0342	524.4289	465.7672	792.3882	673.3309	702.1602	809.8048	769.3905	746.0064
1037.519	993.2181	986.3153	808.3941	662.9767	733.9651	562.8965	523.7957	465.3874	792.3429	672.51	701.5917	808.5641	768.461	745.4037
1042.927	991.4882	986.3183	820.9991	661.8102	733.9673	564.5644	522.8829	465.3888	794.5417	671.3268	701.5938	810.0002	773.1817	745.406
1040.771	993.0337	986.4957	817.6685	662.8524	734.1007	562.274	523.6984	465.4734	791.3184	672.3838	701.7204	806.7142	771.3182	745.5402
1038.557	991.1228	986.1499	819.66	661.5638	733.8408	563.6436	522.6901	465.3086	793.2458	671.0768	701.4737	808.6791	769.8383	745.2787
1046.116	990.5983	983.8195	820.3218	660.1397	732.0888	564.0986	521.5757	464.1977	793.8862	669.6322	699.8109	809.3319	770.2026	743.5161
1048.156	993.7983	983.4135	819.8569	660.6528	731.7836	563.779	521.9772	464.0042	793.4363	670.1527	699.5213	808.8733	770.7919	743.2091
1051.958	998.0074	987.668	815.9821	660.1375	734.982	561.1144	521.5739	466.0322	789.6863	669.6299	702.5568	808.0504	770.2	746.4268
1047.041	997.8248	983.994	816.6648	661.5254	732.22	561.5839	522.66	464.2809	790.3471	671.0378	699.9354	808.7239	771.7941	743.6481
1047.051	994.3475	984.447	818.9857	661.0273	732.5605	563.1798	522.2702	464.4968	792.5931	670.5326	700.2586	808.0137	771.2221	743.9907
1036.55	991.3914	988.4474	818.0624	664.997	737.8232	562.5449	525.3766	467.8337	791.6996	674.5593	705.2534	807.1028	771.7814	742.2852
1048.2	993.8306	987.0224	818.1546	669.4819	742.0143	562.6083	523.8862	470.4912	791.7889	679.1088	702.2311	807.1938	768.9326	746.5017
1043.373	989.6468	986.1201	814.1066	666.4332	738.8163	563.8247	526.5004	468.4634	787.8714	676.0162	703.1959	808.2001	772.4309	743.2843
1036.684	981.899	985.7533	810.8047	662.7673	734.2485	561.5541	523.6318	465.5671	790.6758	672.2975	696.8606	808.9423	768.2205	738.6888
1041.513	997.3636	980.8068	813.873	665.666	737.3417	563.6641	525.9001	467.5284	793.6452	675.238	699.7963	807.9695	771.5498	741.8007
1042.057	995.6747	983.2304	813.3169	666.6539	739.1636	563.2817	526.6732	468.6836	793.1071	676.2401	701.5255	807.4209	772.6845	743.6337
1049.5	997.0154	980.0701	811.7232	665.5372	736.7878	562.1858	525.7993	467.1772	791.5647	675.1073	699.2707	805.8486	771.4018	741.2436
1046.301	992.3239	988.9767	817.456	672.3121	743.4836	562.1279	526.1008	471.8228	791.1128	681.9796	705.6255	809.5045	772.1831	747.9798
1038.44	992.7368	983.8779	804.1224	670.1579	739.6504	560.959	524.4151	468.9923	792.2088	679.7944	701.9875	808.3496	769.709	744.1234
1038.954	997.6335	988.5264	810.6282	671.8664	735.6273	562.4328	525.7521	466.4414	794.5051	681.5275	698.1693	807.7683	771.6713	746.076
1037.058	984.3716	986.3013	809.3804	663.0452	733.9545	561.5747	525.8493	465.3807	793.2975	672.5795	696.5816	808.5372	770.5397	744.3931
1035.02	991.5296	989.7854	802.5648	664.1273	736.5738	560.8879	526.6961	467.0415	791.7015	673.6771	699.0675	809.8129	771.7826	747.0282
1040.078	983.6	980.2905	811.9161	663.0762	736.9536	563.3184	525.8735	467.2823	791.7514	672.6109	699.428	808.0388	770.5753	747.4103
1040.694	994.7924	988.0233	812.2441	663.3316	735.2491	563.5439	526.0734	466.2015	792.0688	672.87	697.8103	808.3625	770.8686	745.6955
1038.065	988.5565	986.9288	816.0203	663.8447	734.4263	561.1406	526.4749	465.6798	795.7233	673.3905	697.0294	809.088	771.458	744.8677
1040.596	997.6662	989.1539	811.9206	665.0858	736.0991	562.3215	527.4461	466.7405	791.7558	674.6494	698.617	808.0433	772.8834	746.5506
1045.505	990.9	987.6176	817.7657	662.0861	742.4619	562.3409	525.0987	470.775	791.4125	671.6065	704.6558	809.8101	769.4381	746.9519
1044.893	988.1161	985.3714	815.3084	658.7885	740.7732	560.6511	522.5183	469.7042	792.0344	678.2615	703.0531	807.3857	771.6506	745.253
1041.539	987.1783	986.751	812.6914	659.2814	741.8103	558.8515	522.904	470.3618	789.5017	672.7615	704.0374	807.8038	772.2167	746.2964
1042.614	995.8895	982.9047	813.5301	659.1458	738.9188	559.4283	522.7979	468.5284	790.3134	673.6239	701.2932	808.6312	772.061	743.3874
1039.277	993.4449	984.4047	810.9259	665.0338	740.0464	557.6375	525.4054	469.2434	787.7931	674.5967	702.3634	808.0619	772.8237	744.5219
1046.13	993.0779	987.0914	816.2737	663.5712	742.0662	561.3149	524.2609	470.5241	792.9686	673.1131	704.2803	808.3381	771.1439	746.5539
1036.646	999.0153	984.2942	808.8734	666.0036	738.9484	556.226	526.1643	468.5472	790.8067	675.5804	701.3213	807.0369	772.9376	743.4172
1040.864	993.8702	986.8982	805.7831	664.1115	742.7218	554.101	522.6837	470.9398	791.8161	673.6611	704.9025	805.9881	770.7644	747.2134
1036.112	987.9839	982.9955	808.4567	666.6471	739.4555	553.9395	521.6678	468.8687	782.4035	674.2332	701.8025	797.6258	765.6766	743.9274
1005.809	957.2608	936.0034	784.812	644.4017	702.3225	539.6801	504.2602	445.3237	759.5207	653.6679	666.5603	774.2978	740.1267	706.5698
990.4138	913.5164	909.4469	772.7993	621.7312	690.0476	531.4195	486.52	437.5405	747.8952	630.6714	654.9105	762.4461	714.0886	694.2207

The measurement for this equipment was made for a pressure range between 1 and 13 bar. According to the applicable legislation, the pressure reading is made at the highest point of the equipment. A linear interpolation has been used in order to compensate the 0–1 bar range. As a consequence the maximum value was augmented by a 1.18 factor. The factor magnitude was obtained by dividing the number of pressure intervals as follows: total incremental intervals (13) by the corresponding number intervals at which the measurement was made (12).

By means of Hooke’s law relationship, one can obtain the principal stresses (*σ*_1_, *σ*_2_) from micro strain (µm/m) values in order to acquire the equivalent von Mises stress (*σ_von Mises_*) according to next Formula:

$$\sigma_{von\;Mises}=\sqrt{\sigma_{1}^{2}+\sigma_{2}^{2}-\sigma_{1}\sigma_{2}}.$$

Table A2 presents the results corresponding to the maximum values recorded for each support leg.

**Table A2:** Computational insight.

Support Leg	Maximum Raw Data (µm/m)			Augmented Values (µm/m)			Principal Stresses (MPa)		Von Mises (MPa)
	1	2	3	1	2	3	*σ*_1_	*σ*_2_	
1	1052.29	998.35	989.81	1136	1078	1069	337.58	324.06	331.03
2	821.09	673.18	744.11	887	727	803	273.71	233.19	255.87
3	564.62	526.78	471.82	610	569	509	176.13	159.53	168.45
4	794.62	682.86	706.22	858	737	762	257.14	228.92	244.26
5	801.08	773.18	748.61	865	835	808	255.59	246.28	251.07

## References

[1] Agbo S., Lin M., Ameli I., Imanpour A., Duan D., Cheng J.J.R., Samer A. (2019). Experimental evaluation of the effect of the internal pressure and flaw size on the tensile strain capacity of welded X42 vintage pipelines. Int. J. Press. Vessel. Pip..

[2] Leis B. Battelle Pipeline. Development Center. Proceedings of the 2009 Government and Industry Pipeline R&D Forum Report.

[3] Wang Y.-Y., Horsley D., Rapp S. Evolution of line pipe manufacturing and its implications on weld properties and pipeline service. Proceedings of the International Pipeline Conference.

[4] Mannucci G., Lucci A., Spinelli C., Baldi A., Mascia G. Full-scale bend testing of strain based designed high grade buried gas pipeline. Proceedings of the Twenty-First International Offshore and Polar Engineering Conference.

[5] Zhu Y., Liang W., Zhao X., Wang X., Xia J. (2019). Strength and stability of spherical pressure hulls with different viewport structures. Int. J. Press. Vessel. Pip..

[6] Takezawa A., Nishiwaki S., Kitamura M., Silva E.C.N. (2010). Topology optimization for designing strain-gauge load cells. Struct. Multidiscip. Optim. J..

[7] Messina M., Njuguna J., Palas C. (2018). Mechanical Structural Design of a MEMS-Based Piezoresistive Accelerometer for Head Injuries Monitoring: A Computational Analysis by Increments of the Sensor Mass Moment of Inertia. Sensors.

[8] Bibbo D., Gabriele S., Scorza A., Schmid M., Sciuto S.A., Conforto S. (2019). A Novel Technique to Design and Optimize Performances of Custom Load Cells for Sport Gesture Analysis. IRBM.

[9] Rosa J., Cagan J., Rosler J. (2017). Strain gauge measurement on small propeller using special in-house developed hardware. Materialstoday Proc..

[10] Bibbo D., Gabriele S., Scorza A., Schmid M., Sciuto S.A., Conforto S. Strain gauges position optimization in designing custom load cells for sport gesture analysis. Proceedings of the IEEE 20th International Conference on E-Health Networking, Applications and Services (Healthcom).

[11] NSAI (2018). EN 13445-3: Unfired Pressure Vessels—Part 3: Design.

[12] NSAI (2016). EN 13445-5: Unfired Pressure Vessels—Part 5: Inspection and Testing.

[13] ASTM (2011). E8/E8M-11 Standard Test Methods for Tension Testing of Metallic Materials.

[14] British Standard Institution (2018). BS 8571: Method of Test for Determination of Fracture Toughness in Metallic Materials Using Single Edge Notched Tension, SENT) Specimens.

[15] Ahmed A.U. (2010). Failure Criteria for Tearing of Telescoping Wrinkles. Ph.D. Thesis.

[16] Wang Y.Y., Cheng W., McLamb M., Horsley D., Zhou J., G A. Tensile strain limits of girth welds with surface-breaking defects—Part 1: An analytical framework. Proceedings of the 4th International Conference on Pipeline Technology.

[17] Robin G., Neil G., Gregory S., Frank R. A comparison of strain-based design ECA models. Proceedings of the Twenty-Seventh International Ocean and Polar Engineering Conference.

[18] Veritas D.N. (January 2006). Fracture Control for Pipeline Installation Methods Introducing Cyclic Plastic, Strain.

[19] Wang Y.Y., Cheng W., McLamb M., Horsley D., Zhou J., Glover A.G. Tensile strain limits of girth welds with surface-breaking defects Part II—Experimental correlation and validation. Proceedings of the 4th International Conference on Pipeline Technology.

[20] Akin J.E. (2009). Finite Element Analysis Concepts via SolidWorks.

[21] SolidWorks User Manual. https://blogs.solidworks.com/solidworksblog/2019/06/solidworks-simulation-accuracy-report-by-afnor.html.

