# Supplementary material for: Use of a Novel Resistive Strain Sensor Approach in an Experimental and Theoretical Study Concerning Large Spherical Storage Tank Structure Behavior During Its Operational Life and Pressure Tests
Source: Sensors (Basel). 2020 Jan 17;20(2):525. doi: 10.3390/s20020525 (PMC7014482; doi:10.3390/s20020525)
Supplement: Supplementary file 1 [file sensors-20-00525-s001.pdf]

Supplementary Materials

Use of a Novel Resistive Strain Sensor Approach in an Experimental and Theoretical Study Concerning Large Spherical Storage Tank Structure Behavior During its Operational Life and Pressure Tests

FEM computational approach - Theory of Elasticity basic knowledge

FEM analysis has been performed using a commercial software solution – Dassault Systèmes SolidWorks[1]. Dassault Systèmes SolidWorks authors do not disclose the FEM mathematical apparatus on which their software is based. However, to give a glimpse of the FEM theory, we present classical equilibrium equations on which FEM simulations are usually based.

As described in Theory of Elasticity (Solid Mechanics) [1, 2] in the case of a plane problem (related to a polar coordinate reference system r,θ), the displacement components u, v are (figure S1, S2):

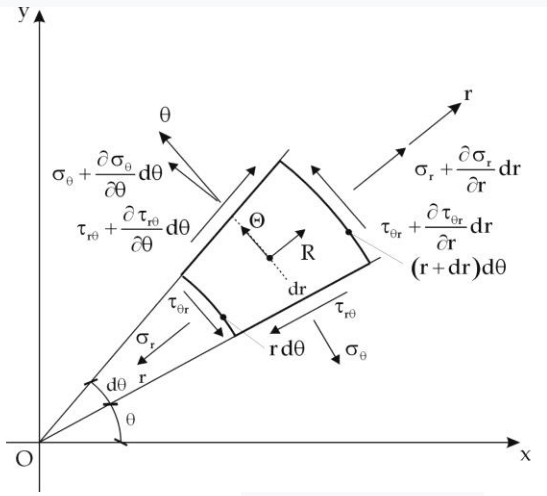

Figure S1 The stress components
Plane discrete element under equilibrium state, related to a polar coordinate reference system

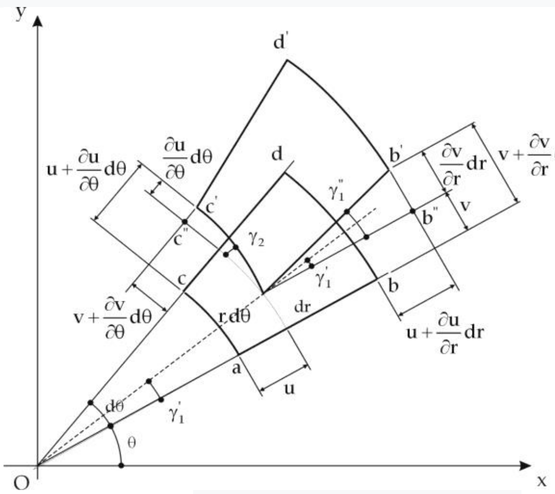

Figure S2 The displacement components
Displacement components in case of a plane discrete element under equilibrium state

Therefore, strain (ε,γ) / displacement (u,v) relations are:

$$\varepsilon_r = \frac{\partial u}{\partial r};$$
$$\varepsilon_\theta = \frac{u}{r} + \frac{1}{r}\frac{\partial v}{\partial \theta}; \qquad \text{(S1)}$$
$$\gamma_{r\theta} = \frac{1}{r}\frac{\partial u}{\partial \theta} + \frac{\partial v}{\partial r} - \frac{v}{r},$$

---

[1] https://www.cadworks.ro/

where $\varepsilon_r, \varepsilon_\theta, \gamma_{r\theta}$ are strain components with respect to polar coordinate system; σ_r, σ_θ are normal stress components and $\tau_{r\theta}, \tau_{\theta r}$ shear stress components, with respect to polar coordinate system.

The strain / stress equations will maintain a plane problem (Cartesian coordinate reference system) structure, with specific strain and stress notation indexes, therefore:

$$\varepsilon_r = \frac{1}{E}\left(\sigma_r - \mu\sigma_\theta\right); \quad \varepsilon_\theta = \frac{1}{E}\left(\sigma_\theta - \mu\sigma_r\right); \quad \gamma_{r\theta} = \frac{2(1+\mu)}{E}\tau_{r\theta}, \tag{S2}$$

with μ - Poisson's ratio (the ratio of transverse contraction strain to longitudinal extension strain, in the direction of axial force) .

If both loads and boundary conditions can be described as axisymmetric (figure S3), static study equations become:

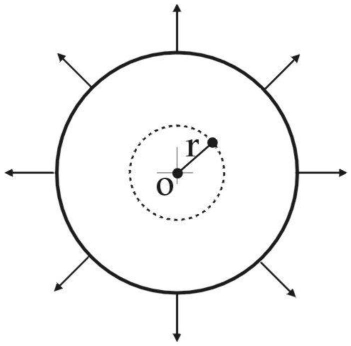

Figure S3 The loads and boundary conditions
Example of an axisymmetric equilibrium state plane element

$$\frac{d\sigma_r}{dr} + \frac{1}{r}\left(\sigma_r - \sigma_\theta\right) = 0, \tag{S3}$$

or, in strain terms point of view:

$$\varepsilon_r = \frac{du}{dr}; \quad \varepsilon_\theta = \frac{u}{r}; \quad \gamma_{r\theta} = 0, \tag{S4}$$

where:

$$\varepsilon_r = \frac{1}{E}\left(\sigma_r - \mu\sigma_\theta\right); \quad \varepsilon_\theta = \frac{1}{E}\left(\sigma_\theta - \mu\sigma_r\right). \tag{S5}$$

The axisymmetric plane state of stress (figure S4), has a significant effect on the stress/strain matrix equation, therefore the generalized Hooke's law is:

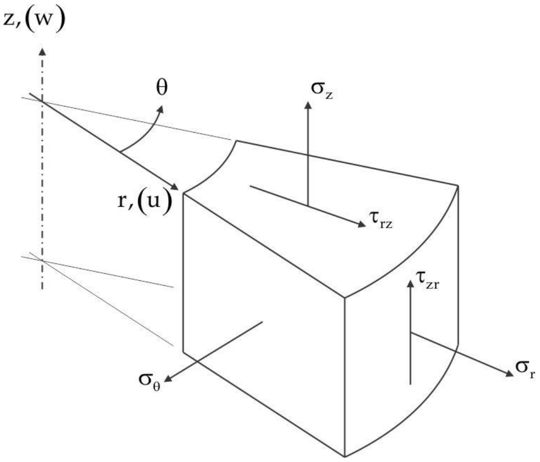

Figure S4 The axisymmetric plane state of stress
Example of an axisymmetric equilibrium state volume element, with respect to a polar coordinate system

$$\{\sigma\} = [D] * \{\varepsilon\}, \tag{S6}$$

where $\{\sigma\}$ represents the stress array, $\{\varepsilon\}$ the strain array and $[D]$ the elasticity matrix, in polar coordinate system symbols:

$$\begin{Bmatrix} \sigma_r \\ \sigma_\theta \\ \sigma_z \\ \tau_{rz} \end{Bmatrix} = \begin{bmatrix} \frac{(1-\mu)E}{(1+\mu)(1-2\mu)} & \frac{\mu E}{(1+\mu)(1-2\mu)} & \frac{\mu E}{(1+\mu)(1-2\mu)} & 0 \\ \frac{\mu E}{(1+\mu)(1-2\mu)} & \frac{(1-\mu)E}{(1+\mu)(1-2\mu)} & \frac{\mu E}{(1+\mu)(1-2\mu)} & 0 \\ \frac{\mu E}{(1+\mu)(1-2\mu)} & \frac{\mu E}{(1+\mu)(1-2\mu)} & \frac{(1-\mu)E}{(1+\mu)(1-2\mu)} & 0 \\ 0 & 0 & 0 & \frac{E}{2(1+\mu)} \end{bmatrix} * \begin{Bmatrix} \varepsilon_r \\ \varepsilon_\theta \\ \varepsilon_z \\ \gamma_{rz} \end{Bmatrix}, \tag{S7}$$

the matrix equation system (S7) being used by finite element method based software packages.

As mentioned before, we did our FEM simulations using SolidWorks software, SolidWorks Simulation Linear Static "*analysis calculates displacement, strains, stresses and reaction forces under the effect of applied loads – the sequence of calculation is composed by several compulsory steps such as construction and solving a system of linear simultaneous finite element equilibrium equations (mesh, material properties, restrains and loads), in order to obtain displacement components at each node. Three direct solvers and one iterative solver are available for the solution of the set of equation. In FEM analysis, a problem can be represented by a set of algebraic equations that must be solved simultaneously. There are two classes of solution methods, direct and iterative; direct methods solve the equations using exact numerical techniques. Iterative methods solve the equation using approximate techniques where in each iteration, a solution is assumed and the associated errors are evaluated; the iterations continue until the errors become acceptable. Based on displacement field one can reach the strain components and finally, the program uses the strain results and the stress/strain relationships to calculate the stresses. Gauss-Legendre computational approach is used in finite element code. Stress results are first calculated at special points (Figure 5), called Gaussian points or Quadrature points, located inside each element. In numerical analysis, a quadrature rule*

is an approximation of the definite integral of a function, stated as a weighted sum of function values at specified points within the domain of integration.

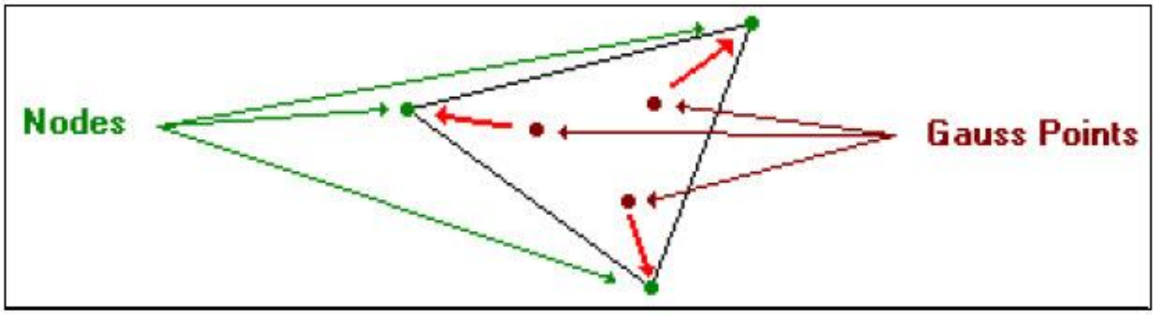

Figure S5 – Gaussian points versus nodal points placement

These points are selected to give optimal numerical results. The program calculates stresses at the nodes of each element by extrapolating the results available at the Gaussian points. After a successful run, nodal stress results at each node of every element are available in the database" (SolidWorks Simulation user manual).

One can assess the scatter plot of F.E.M. specific mathematical apparatus output measurements (D'Assault Systemes SolidWorks particular case) versus traditional computing approach, as suggested by a number of comparative study cases [3, 4, 5], for instance stiffness-related structural optimisation [5], Figure S6.

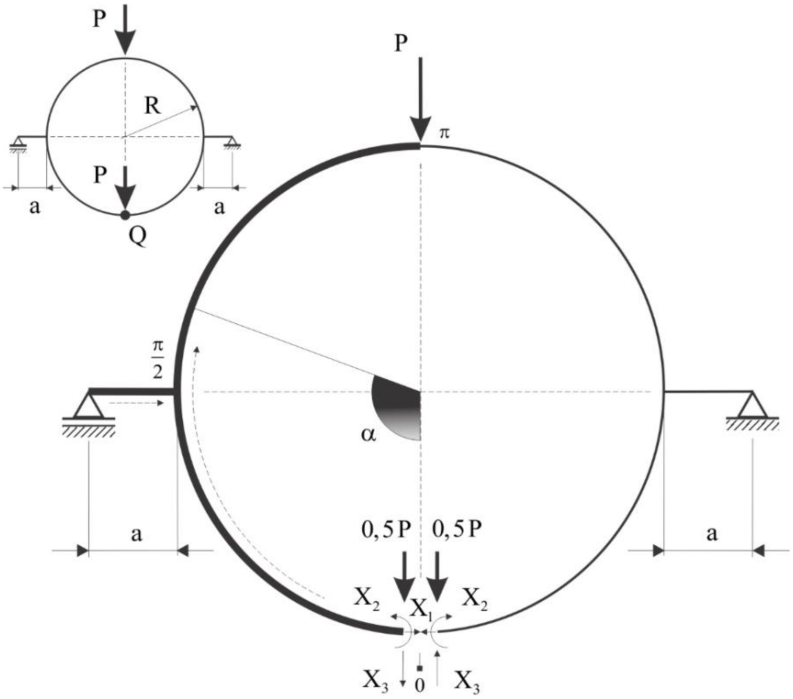

a. *Support and loading scheme for a statically indeterminate plane frame structure*

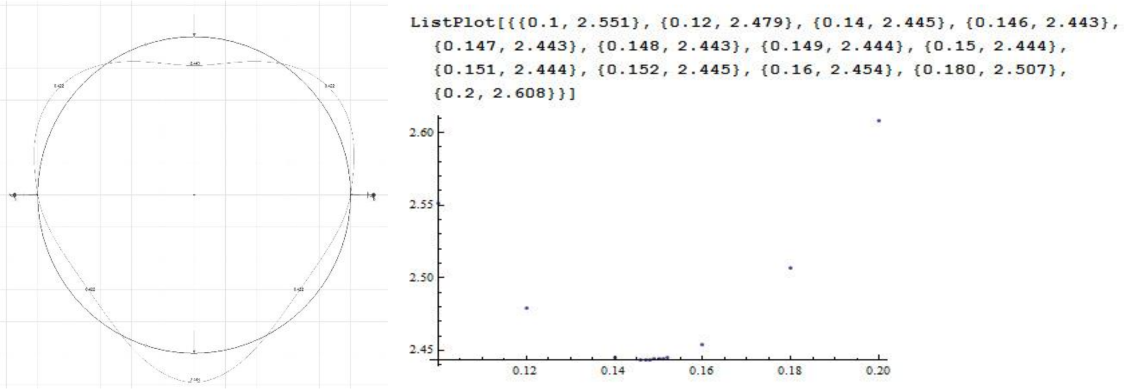

b. *Vertical displacement component field – AxisVM vs.traditional approach*

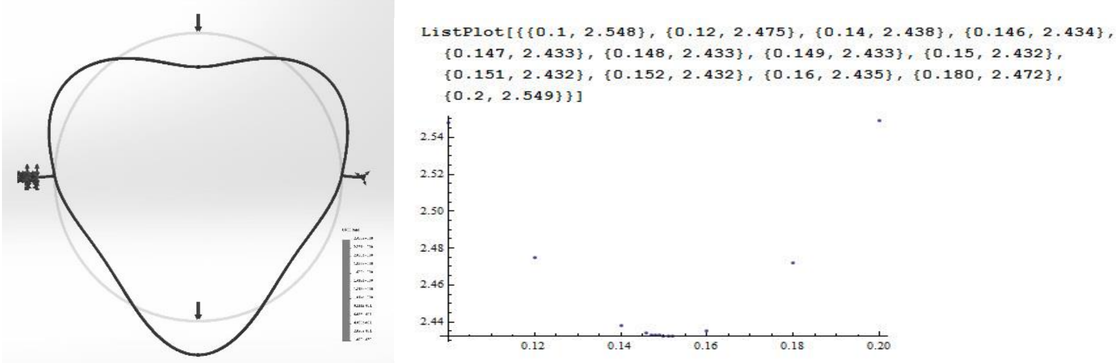

c. *Vertical displacement component field – SolidWorks 2014 vs.traditional approach*

Figure B6 The output measurements versus traditional computing approach

In Figure S6 one can assess the excellent scatter plot of output measurements (vertical displacement component field), obtained via a dual-approach example concerning traditional calculus method [5], versus Finite Element Method (FEM) analysis for a statically indeterminate plane frame structure.

References

1. N.I. Bezuhov, Theory of Elasticity and Plasticity (in Romanian), Editura Tehnica , Bucuresti, **1957**;
2. St. Mocanu, Complements to the Theory of Elasticity (in Romanian), University of Civil Engineering of Bucharest, **2007**, http://fem.utilajutcb.ro/auxiliare/complemente-de-Teoria-Elastici/complemente-de-teoria-elasticitatii.html;
3. St. Mocanu, Short Comment with Reference to the Balance and Strain/Stress State for a Straight Rod of Circular Section Subjected to Torsional Load (in Romanian). In Syntheses of Theoretical and Applied Mechanics, pp. 69-73, vol.5, no.1, Bucureşti: Ed.MatrixRom., **2014**, ISSN 2068 – 6331.
4. St.Mocanu, Aspects Regarding the Use of Numerical Methods when Modeling the Behavior of the Structures Required for Lateral Buckling (in Romanian). In Syntheses of Theoretical and Applied Mechanics, pp. 5-14, vol. 4, no.1, Bucureşti: Ed.MatrixRom, **2013**, ISSN 2068 – 6331.
5. St. Mocanu, Mechanical Stiffness as an Optimization Parameter for a given Structure (in Romanian). In Syntheses of Theoretical and Applied Mechanics, pp. 335-342, vol.7, no.4, Bucureşti: Ed.MatrixRom, **2016**, ISSN 2068.
